# First exposure to second-generation antipsychotics alters gut microbiota and metabolic profiles in patients with glucose-lipid metabolism disorders

**DOI:** 10.3389/fpsyt.2025.1722760

**Published:** 2025-12-08

**Authors:** Yuexia Wu, Sai Zang, Zhiming Wu, Jingsong Huang

**Affiliations:** 1 Department of Psychiatry, Dalian Seventh People’s Hospital, Dalian, China; 2 Department of Child Health Care, Xi’an Children’s Hospital, Xi’an, China; 3 Graduate School of Dalian Medical University, Dalian, China

**Keywords:** schizophrenia, antipsychotic, gut microbiota, metabolomic analysis, glucose and lipid metabolism

## Abstract

**Backgroud:**

Second-generation antipsychotics (SGAs) are widely used in the treatment of schizophrenia, however, growing concerns have emerged regarding their adverse effects on glucose and lipid metabolism. This study aimed to investigate the potential mechanisms underlying SGA-induced disturbances in glucose and lipid metabolism by integrating gut microbiota profiling with metabolomic analysis, thereby providing a scientific basis for future clinical interventions.

**Methods:**

A self-controlled before-after study was conducted on subjects who were initially medication-free (pre-medication group) and subsequently initiated on second-generation antipsychotics for 3 months (post-medication group). Anthropometric measurements—including waist circumference, hip circumference, body weight—as well as fasting blood samples (for assessment of glucose, insulin, C peptide, blood lipid) and stool samples were collected at baseline and after three months of treatment. Gut microbiota composition and fecal metabolomic profiles were analyzed using high-throughput sequencing and mass spectrometry–based approaches, respectively.

**Results:**

Firstly, Gut microbial diversity differed significantly between groups. At genus level, the abundances of *Escherichia* and *Bifidobacterium* were increased significantly in the post-medication patients, while the abundances of *Faecalibacterium* and *Blautia* were decreased. Metabolomic analysis revealed decreased levels of oleamide and stearamide in the post-medication group, which exhibited a negative correlation with the abundance of *Faecalibacterium*. Additionally, the arginine and proline metabolic pathway, D-amino acid metabolic pathway, and arginine biosynthesis pathway were also altered in this group. Ornithine was identified as a key player in these three differential metabolic pathways.

**Conclusion:**

In summary, first-time exposure to second-generation antipsychotics in patients with schizophrenia is associated with disturbances in glucose and lipid metabolism, which appear to be closely linked to SGA-induced perturbations in gut microbiota composition and their associated metabolic profiles.

## 1 Introduction

With the widespread application of SGAs, increasing amounts of psychiatric patients are experiencing significant weight gain, hypercholesterolemia, hypertension, and diabetes—conditions collectively implicated in metabolic syndrome and cardiovascular disease (1). Current epidemiological studies show that the prevalence of metabolic syndrome among individuals with schizophrenia ranges from 30% to 40% (2, 3), contributing to a reduction in life expectancy by 10–20 years and substantially exacerbating the global burden of mental illness (4). In recent years, more and more scholars have devoted themselves to the study of antipsychotic-induced metabolic dysfunction (AIMD). However, the pathogenesis of antipsychotics disturbances in glucose and lipid metabolism remains incompletely understood.

Although existing evidence suggests that pathogenesis of abnormalities of glucose and lipid metabolism is influenced by insulin resistance, dopamine reward system regulation disorders, lifestyle, etc. (5–7), emerging research highlights the gut microbiota as a pivotal mediator in AIMD. Morgan et al. established a mouse model and proved that gut microbiota was a necessary condition for AIMD (8). In the experiment, high-fat diet (HFD) mice administered with olanzapine for 7 consecutive weeks exhibited greater weight gain than those on HFD alone; however, this effect was abolished in germ-free mice, where no significant difference in weight gain was observed between olanzapine-treated and control groups. Subsequent experiments further revealed that olanzapine reduced alpha diversity of the gut microbiota, whereas aripiprazole significantly increased it (8, 9). Li-Ya Pan et al. discovered that juvenile patients who developed weight gain after three months of atypical antipsychotic (AP) treatment showed marked increases in the relative abundance of *Romboutsia* and *Klebsiella (*10). Moreover, metformin has been shown to alleviate AIMD by enriching SCFA-producing gut microbiota—such as *Bifidobacterium* and *Butyricimonas—*via the enterohepatic axis (11, 12).

Despite these advances, research on gut microbiota dysbiosis in the context of AIMD remains in its early stages, with the majority of evidence derived from animal models. Human studies are comparatively scarce. Most published clinical investigations have relied on 16S rRNA gene sequencing to identify compositional differences in the gut microbial community but have not comprehensively characterized the associated metabolic alterations. To address this gap, the present study employed untargeted metabolomic profiling of stool samples to identify key differential metabolites and dysregulated metabolic pathways linked to AIMD, thereby offering deeper mechanistic insights into the role of the gut microbiome in antipsychotic-induced metabolic disturbances.

## 2 Methods

### 2.1 Clinical patients

A total of 30 patients diagnosed with schizophrenia and admitted to *Dalian Seventh People’s Hospital* from June 2022 to November 2023 were recruited into this study. All participants were antipsychotic-naive and initiated treatment with SGAs for the first time. A self-controlled before-after study was conducted, comparing each participant’s baseline status prior to medication initiation (pre-medication group, B group) with their condition after three months of SGA treatment (post-medication group, M group). The specific SGA prescribed was determined by the attending physician based on individual clinical considerations. This study was approved by the Ethics Committee of Dalian Seventh People’s Hospital (approval number: 2021-09). All participants were informed about the study procedures and potential risks and signed an informed consent form.

Inclusion criteria: (1) The patient met the DSM-5 diagnostic criteria for schizophrenia, with a total PANSS score of ≥60 points; (2) No gender limit, age between 18 and 45 years old;(3) Blood routine, heart, liver, and kidney functions were normal; (4) Patients should have an education level of primary school or above, have good understanding, and be able to cooperate and successfully complete the research.

Exclusion criteria: (1) Use of antipsychotics, antibiotics, intestinal antispasmodics, or any other drugs known to affect gut microbiota within three months before enrollment; (2) Presence of organic brain disease, coronary heart disease, hypertension, diabetes mellitus, hepatic disease, metabolic disorders, obesity, or other comorbid neuropsychiatric diseases; (3) Pregnancy or lactation.

### 2.2 Blood biochemical indicator measurement

At enrollment and after 3 months of SGAs treatment, anthropometric measurements—including waist circumference (WC), hip circumference, and body weight—were recorded. Fasting blood and stool samples were also collected at both time points. Freshly collected fecal specimens were immediately frozen in a refrigerator at -80 °C. Serum fasting blood glucose (FBG) was measured using the hexokinase method. Total cholesterol (TC) and triglycerides (TG) were determined by the cholesterol oxidase method (CHOD-PAP) and the glycerol phosphate oxidase method (GPO-PAP), respectively. High-density lipoprotein (HDL) and low-density lipoprotein (LDL) were determined by the direct homogeneous method. Fasting insulin and C peptide levels were determined by enzyme-linked immunosorbent assay (ELISA). The homeostatic model assessment of insulin resistance (HOMA-IR) was calculated using the formula: HOMA-IR = fasting blood glucose (FBG, mmol/L) × fasting insulin (FINS, mIU/L)/22.5, which was used to assess the insulin sensitivity and insulin resistance levels of the study subjects.

### 2.3 16S rRNA gene amplicon sequencing and data handling

DNA extraction from clinical samples and 16S rRNA data analysis were performed by Wekemo Bioincloud (Guangzhou, China).

The DADA2 plugin in Qiime2 software performed quality control, denoising, merge, and de-chimerization on all raw sequences from every sample to generate amplifying signature sequences (ASVs), also known as feature sequences. ASVs representative sequences were selected and aligned against the Greengenes database (version 13_8) to obtain species annotation information. Linear Discriminant Analysis Effect Size (LEfSe) and Analysis of composition of microbiomes (ANCOM) analysis were performed to assess intergroup differences at various taxonomic levels (species).

Alpha diversity was assessed according to observed-features, Chao1, Shannon indices to evaluate the species diversity of samples. Differences in species diversity between the two groups were calculated via Wilcoxon signed-rank test (two-tailed). Beta diversity was estimated by Partial Least Squares Discriminant Analysis (PLS-DA) analysis and Non-metric multidimensional scaling (NMDS) analysis. PLS-DA coordinate plots were used to assess the effectiveness of the classification model between groups, and NMDS better reflected the nonlinear structure of ecological data.

### 2.4 Measurement of metabolites

Untargeted metabolomics analysis was applied to compare the structural differences of metabolites, screen characteristic metabolites and analyze metabolic pathways. Briefly, 300 μL of 80% aqueous methanol was added to each sample. The mixture was immediately snap-forzen in liquid nitrogen for 5 minutes, thawed on ice, vortexed for 30s, and then sonicated for 6min. Then the samples were centrifuged at 5,000 rpm, 4°C for 1 min. The supernatant was transferred to a new centrifuge tube and lyophilized to dryness. Finally, the resulting residue was reconstituted in an appropriate volume of 10% methanol solution based on the initial sample amount and subjected to liquid chromatography–mass spectrometry (LC–MS) analysis (13, 14). The metabolomic data was processed using the MetaboAnalyst R package. Prior to bioinformatic analysis, quality control, batch effect correction, and data normalization were performed to ensure analytical reliability and comparability across samples.

### 2.5 Sample size and power

This study was designed as a pilot investigation to identify potential microbial and metabolic signatures associated with initial SGA exposure. A *post hoc* power analysis indicated that with n = 15, the study had approximately 80% power to detect large effect sizes (Cohen**’**s d ≥ 0.8) but limited power (<60%) for moderate effects (d ≈ 0.5) at α = 0.05.

### 2.6 Statistical considerations and power analysis

Normality of continuous variables was assessed using the Shapiro–Wilk test (all *P* > 0.05). Paired *t*-tests (two-tailed) were used for normally distributed data, while non-normally distributed microbial α-diversity indices were compared using the Wilcoxon signed-rank test. For multiple comparisons in microbiome and metabolomic analyses, false discovery rate (FDR) correction was applied where feasible; however, due to the exploratory nature and limited sample size (*n* = 15), uncorrected *P* values are reported with caution, and findings should be considered hypothesis-generating. Effect sizes were calculated as Cohen’s *d* for paired comparisons and Spearman’s ρ for correlation analyses. All statistical tests were two-sided, and *P* < 0.05 was considered statistically significant unless otherwise specified. Analyses were performed using R (v3.4.3), Python (v2.7.6), GraphPad Prism (v9.0), and CentOS (CentOS release 6.6). The identified metabolites were annotated by the KEGG database (https://www.genome.jp/kegg/pathway.html), the HMDB database (https://hmdb.ca/metabolites) and the LIPID Maps database (http://www.lipidmaps.org/).

## 3 Result

### 3.1 Comparison of clinical data

After excluding 5 patients who did not provide a stool sample, 8 patients who were lost to follow-up, and 2 patients who failed to adhere to the prescribed medication regimen, a total of 15 patients (8 males and 7 females) were recruited in the final analysis. The data met the assumption of normality (Shapiro-Wilk test, *P* > 0.05). Compared with the pre-medication group (B group), the post-medication group (M group) exhibited significantly higher levels of FBG, FINS, C-peptide, IR index, TC and TG values (*P* < 0.05, paired *t*-test). The WHR value was markedly elevated in M group (*P* < 0.01, paired *t*-test), and the BMI and WC values in the M group showed substantial increases (*P* < 0.001, paired *t*-test) (**Table 1**).

**Table 1:** Comparison of clinical data (Mean ± SD, n = 15).

Index	B	M	t	P
BMI (kg/m^2^)	20.21 ± 2.85	22.34 ± 2.81	-5.129	<0.001
WC (cm)	75.52 ± 9.46	81.50 ± 8.87	-8.488	<0.001
WHR	0.83 ± 0.05	0.85 ± 0.05	-3.110	0.008
FBG (mmol/L)	4.80 ± 0.49	5.81 ± 1.34	-2.318	0.036
FINS (mIU/L)	16.80 ± 6.87	21.04 ± 5.81	-2.164	0.048
C-peptide (ng/mL)	0.25 ± 0.18	0.93 ± 0.93	-2.824	0.014
IR	3.58 ± 1.47	5.50 ± 2.16	-2.938	0.011
TC (mmol/L)	3.93 ± 0.62	4.62 ± 1.38	-2.209	0.044
TG (mmol/L)	1.01 ± 0.50	1.72 ± 1.14	-2.261	0.040
HDL (mmol/L)	1.19 ± 0.24	1.11 ± 0.25	1.277	0.222
LDL (mmol/L)	2.62 ± 0.59	3.04 ± 1.12	-1.619	0.128

Comparisons were performed using paired *t*-tests (two-tailed). Effect sizes (Cohen’s *d*) for significant comparisons: BMI (1.32), WC (2.19), WHR (0.80), FBG (0.60), FINS (0.56), C-peptide (0.73), IR (0.76), TC (0.58), TG (0.61). Abbreviation: BMI, body mass index; WC, waist circumference; WHR, waist-to-hip ratio; FBG, fasting blood glucose; FINS, fasting insulin; IR, insulin resistance; TC, total cholesterol; TG, triglycerides; HDL, high density lipoprotein cholesterol; LDL, low-density lipoprotein cholesterol.

### 3.2 Gut microbiota profiling in research participants

#### 3.2.1 Diversity of gut microbiota

**Figure 1:**
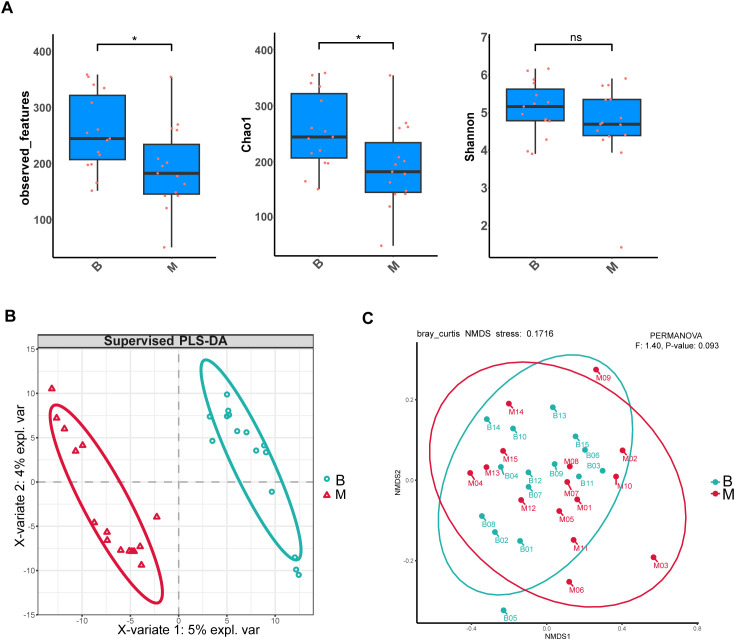
Alterations in gut microbial diversity following SGA treatment. **(A)** α-Diversity indices (observed features, Chao1, Shannon) are presented as box plots showing median, interquartile range, and outliers. Comparisons between B and M groups were performed using the Wilcoxon signed-rank test (two-tailed); *P* < 0.05 was considered significant (*P < 0.05; ns: not significant). **(B)** PLS-DA score plot based on genus-level relative abundances. Ellipses denote 95% confidence regions for each group. **(C)** NMDS plot of Bray–Curtis dissimilarities. Group separation was evaluated by PERMANOVA: *F* = 1.40, *P* = 0.093.

There were significant differences in the α-diversity of gut microbiota between the B and M groups. Specifically, both the observed features and Chao1 indices—metrics reflecting microbial richness—were markedly reduced in Group M compared to Group B. In contrast, the Shannon index, which accounts for both richness and evenness, showed no significant difference between the two groups (**Figure 1A**). With regard to β-diversity, PLS-DA coordinate plot showed a remarkable difference in microbial abundance between B and M groups (**Figure 1B**). In addition, NMDS analysis based on Bray-Curtis dissimilarity revealed distinct clustering of microbial communities between the two groups (stress < 0.2) (**Figure 1C**). Collectively, these results indicate that SGA treatment was associated with significant alterations in both the richness and overall structure of the gut microbiota.

#### 3.2.2 Composition of gut microbiota

**Figure 2:**
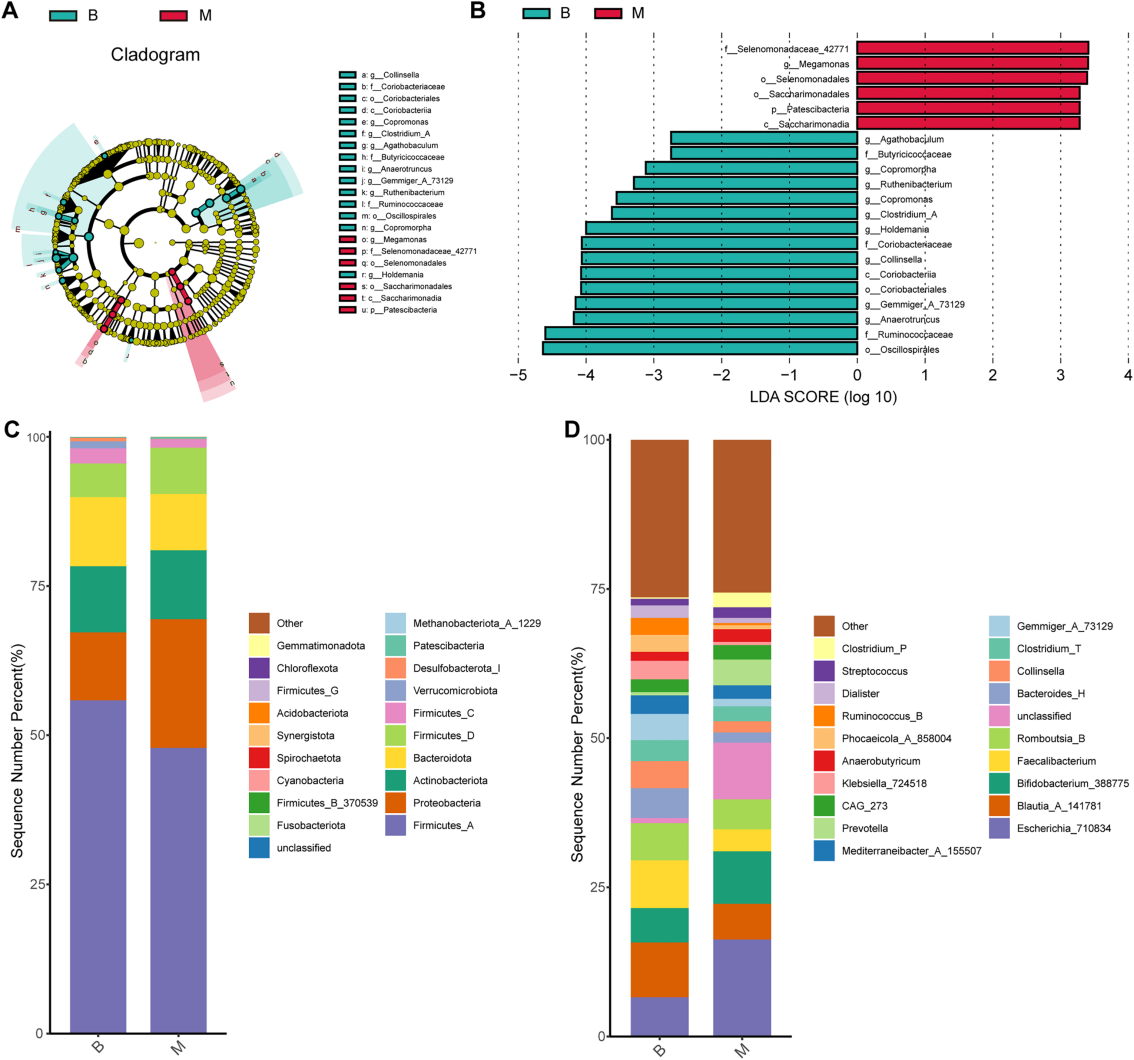
Differences in composition of gut microbiota in each group at genus and phylum levels. **(A)** LEfSe cladogram showing taxonomic hierarchy; colored nodes indicate genera significantly enriched in B (green) or M (red) groups. **(B)** LDA scores (>2.0) from LEfSe analysis. **(C, D)** Stacked bar plots showing relative abundances at phylum **(C)** and genus **(D)** levels (top 10 taxa shown).

ANCOM was employed to assess differential abundance of microbial taxa between groups, revealing significant differences at the genus level (**Supplementary Figure 1A**). LEfSe was further conducted to identify potential microbial biomarkers at genus levels between the two groups. Six genera—including *Selenomonadaceae_42771*, *Megamonas*, *Selenomonadales*, *Saccharimonadales*, *Patescibacteria*, and *Saccharimonadia*—were significantly enriched in the M in comparison with the B group (**Figures 2A, B**). In contrast, 15 genera—including *Agathobaculum*, *Butyricicoccaceae, Copromorpha, Ruthenibacterium, Copromonas, Clostridium_A, Holdemania, Coriobacteriaceae, Collinsella, Coriobacteriia, Coriobacteriales, Gemmiger_A_73129, Anaerotruncus, Ruminococcaceae*, and *Oscillospirales*—showed higher relative abundance in B group than in M group (**Figures 2A, B**). DESeq2-based differential abundance analysis at the genus level further indicated that overall microbial abundance was higher in Group B than in Group M, a finding consistent with the LEfSe results (**Supplementary Figure 1B**, log2FoldChange = 2).

At the phylum level, the four most numerous phyla were *Firmicutes_A*, *Proteobacteria*, *Actinobacteria*, and *Bacteroidota* in both B and M groups (**Figure 2C**). At the genus level, although no statically significant differences were observed across the entire community, notable shifts in specific taxa were eveident. *Blautia_A_141781* was the most abundant genus in B group, with a relative abundance of 9.17%, whereas *Escherichia_710834* predominated in M group (16.30%). Additionally, *Bifidobacterium_388775*, *Faecalibacterium*, and *Romboutsia_B* were among the relatively abundant genera in their respective groups (**Figure 2D**).

### 3.3 Metabolomic profiling of intestinal flora

QA, QC, and data standardization were employed to assess the reliability and comparability of the samples. PCA analysis results indicated that the sample calibration was effective (**Supplementary Figures 2A, B**). Prior to normalization, metabolite intensities exhibited considerable variability in median values and interquartile ranges across samples. After normalization, however, these distributions converged markedly, with medians and quartiles aligning closely across the dataset (**Supplementary Figure 2C**).

#### 3.3.1 Screening of marked differential metabolites

**Figure 3:**
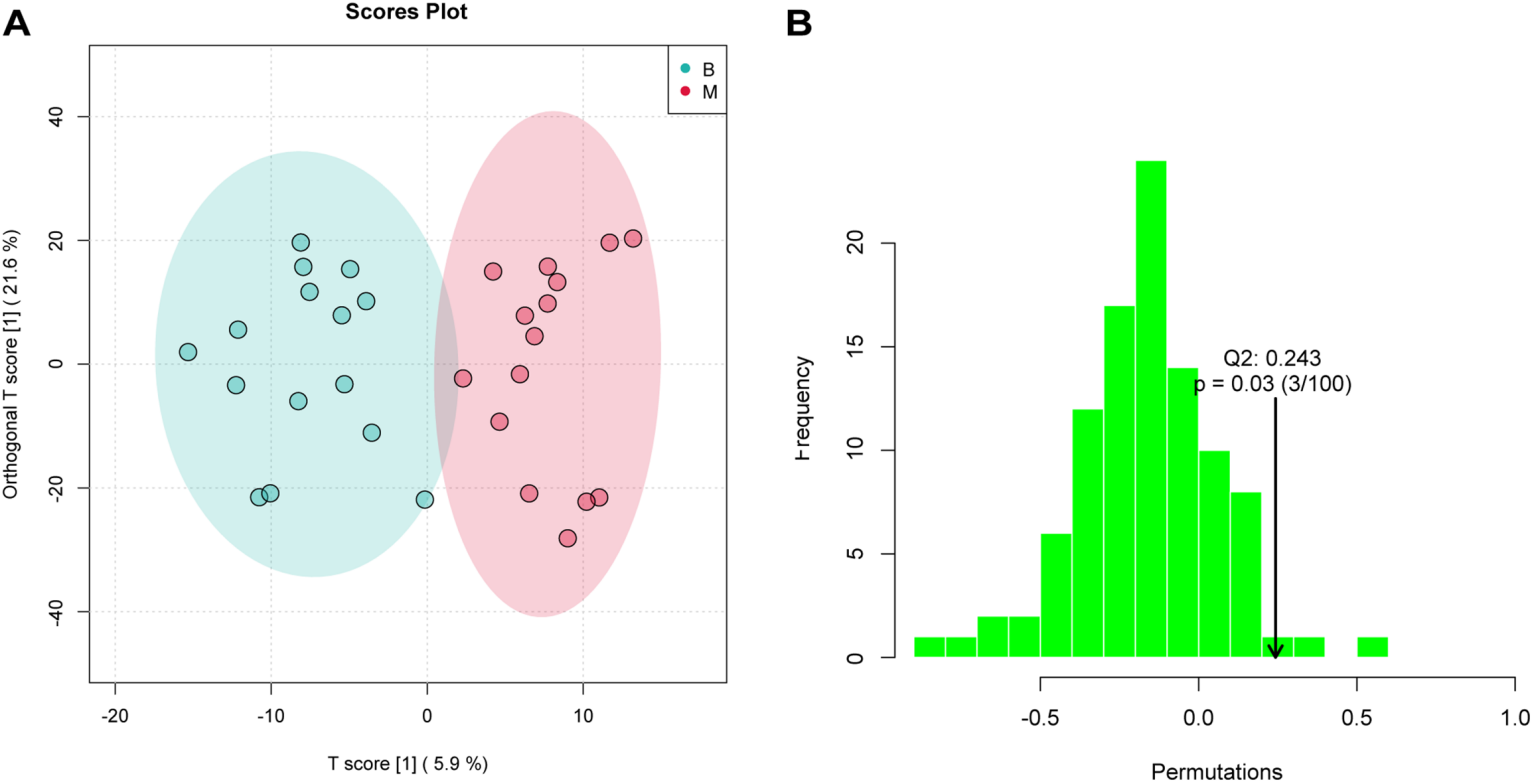
OPLS-DA analysis assessed significant differences of metabolites. **(A)** OPLS-DA score plot showing clear separation between B and M groups (R²Y = 0.838, Q² = 0.243). Ellipses represent 95% confidence regions. **(B)** Permutation test (n = 30 permutations) validating model robustness; the observed Q² (arrow) is significantly higher than the null distribution (*P* = 0.03), indicating no overfitting.

An orthogonal partial least squares–discriminant analysis (OPLS-DA) model was constructed to explore the association between metabolite expression profiles and group assignment (Group B vs. Group M). In this approach, a single predictive component was used to capture variation related to group separation, while all other orthogonal components modeled variation unrelated (i.e., orthogonal) to the class distinction. The OPLS-DA score plot revealed clear separation between Group B and Group M, with sample point clouds occupying distinct regions of the latent space (**Figure 3A**), indicating strong discriminative performance of the model and suggesting substantial metabolic differences between the two groups.

In the permutation test for OPLSDA, we used Q2 [1 - (model error variance/total model variance)] as the test statistic and obtained the random distribution of Q2 through permutation methods. As shown in **Figure 3B**, the observed Q2 value (indicated by the arrow) lay far to the right of the null distribution generated from permuted data, demonstrating that the model’s predictive ability was significantly greater than chance (i.e., non-random). This result supports the presence of genuine differential metabolites between Group B and Group M.

#### 3.3.2 Metabolite content statistics

**Figure 4:**
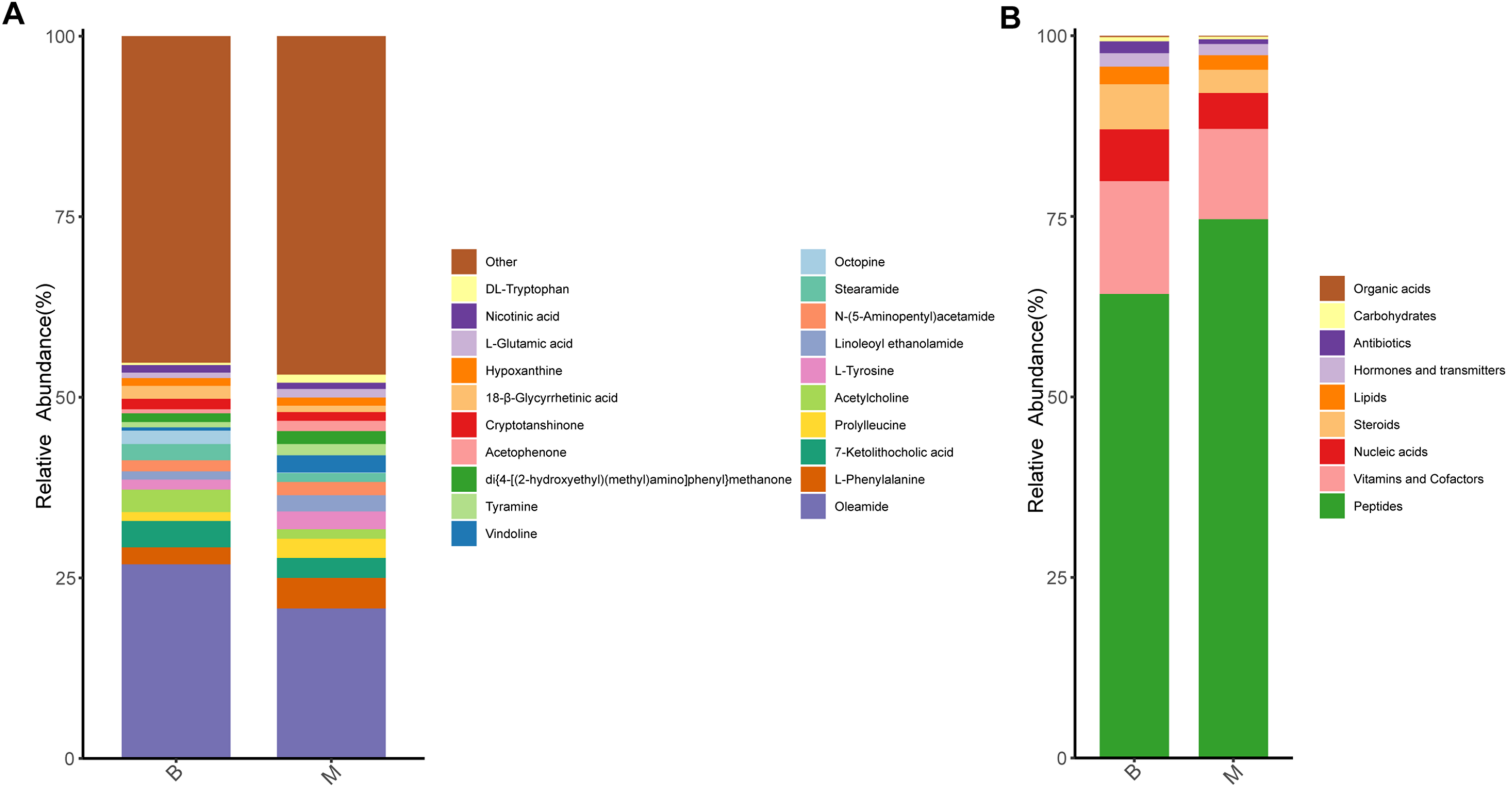
Analysis of metabolite content in B and M groups. **(A)** Relative abundance of the top 20 metabolites in the two groups (stacked bar chart). **(B)** Functional classification of detected metabolites using KEGG BRITE (br08001); major categories include peptides, vitamins/cofactors, nucleic acid derivatives, and steroids.

Differences in metabolite composition and relative abundance between groups were visually assessed by calculating and visualizing the percent content of each metabolite within individual samples. A total of 1,346 metabolites were identified across all samples in this study. The findings showed the relative abundance of top 20 metabolites in each group. As shown in the stacking bar chart, the relative contents of oleamide, 7-ketolithocholic acid, acetylcholine, stearamide and octopine were markedly lower in the M group compared with the B group. Conversely, the relative abundance of L-phenylalanine, prolylleucine, L-tyrosine, linoleoyl ethanolamide and N-(5-Aminopentyl)acetamide significantly increased in M group (**Figure 4A**).

Given that certain metabolites serve defined biological functions—such as hormones, neurotransmitters, or vitamins—we annotated all detected microbial metabolites using KEGG BRITE functional hierarchy (br08001) to categorize their putative biological roles. The majority of annotated metabolites were classified as peptides (64.2% in B group; 74.6% in M group), followed by vitamins and cofactors, nucleic acid derivatives, and steroids (**Figure 4B**).

### 3.4 Metabolic pathway and correlation analysis

**Figure 5:**
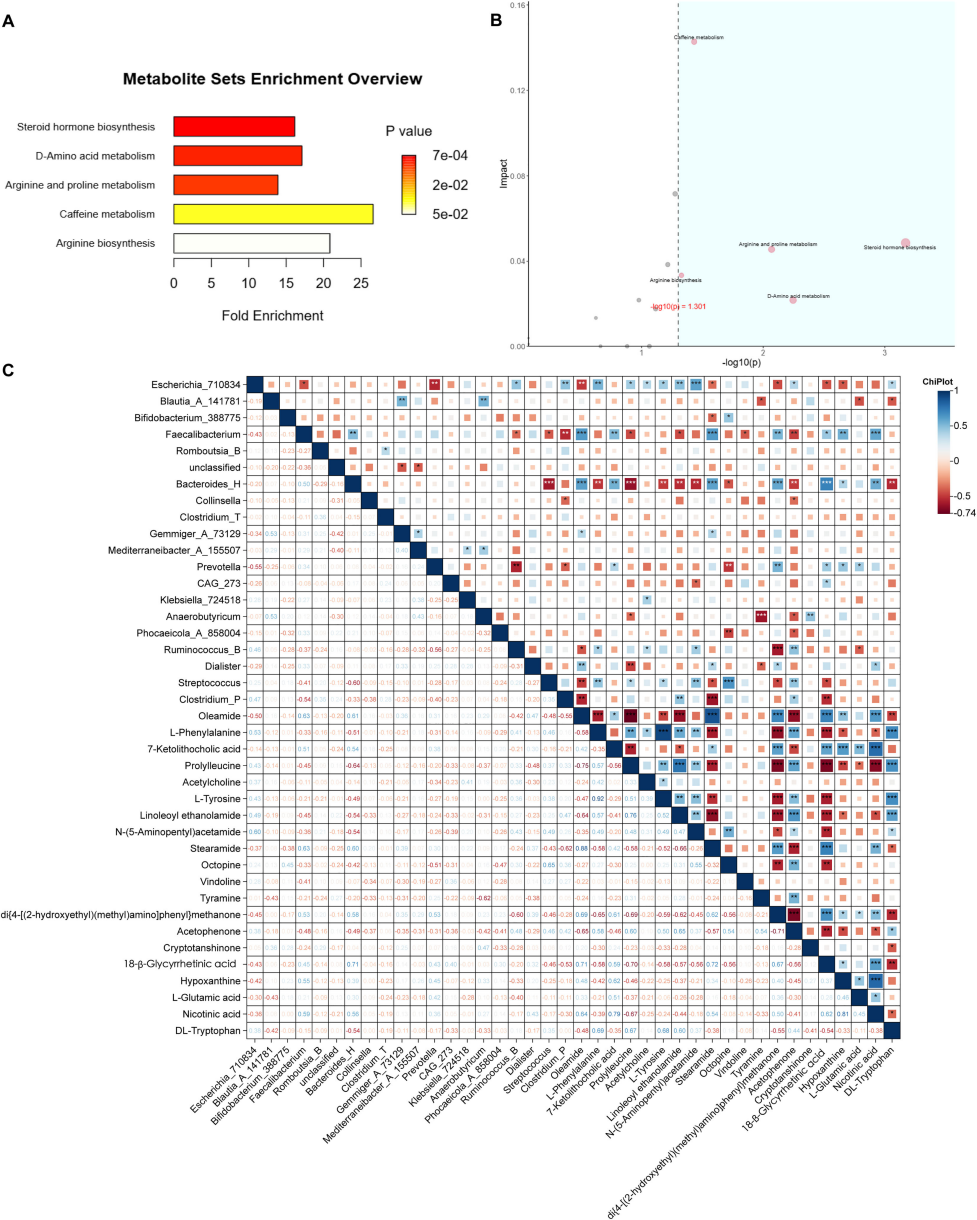
Integration of metabolomic and microbial data. **(A)** ORA of differential metabolites; horizontal axis: enrichment factor. **(B)** Pathway topology analysis combining ORA *P* values [horizontal, –log_10_(p)] and pathway impact (vertical); pathways with *P* < 0.05 and impact > 0.10 are highlighted in blue. **(C)** Spearman correlation heatmap between key genera and metabolites analyzed by Chiplot. Only correlations with |ρ| > 0.6 and *P* < 0.05 are displayed (*P < 0.05; **P < 0.01; ***P < 0.001). No correction for multiple testing was applied due to the exploratory design.

Enrichment analysis of metabolic pathways was performed for the identified differential metabolites. Both over-representation analysis (ORA) and topological pathway analysis indicated that these metabolites were significantly enriched in four key metabolic pathways: amino acid metabolism, arginine and proline metabolism, D-amino acid metabolism, and arginine biosynthesis (**Figures 5A, B**).

Meanwhile, a correlation analysis between gut microbiota and metabolites abundances was conducted using ChiPlot. The results revealed strong positive or negative associations between specific metabolites and bacterial genera. Notably, N-(5-Aminopentyl)acetamide and linoleoyl ethanolamide exhibited significant correlation with *Escherichia_710834*. Oleamide, 7-ketolithocolic acid, stearamide, di{4-[(2-hydroxyethyl)(methyl)amino]phenyl}methanone, 18β-glycyrrgetinin acid, hypoxanthine and nicotinic acid were strongly correlated with *Faecalibacterium* and *Bacteroides_H*. Additionally, di{4-[(2-hydroxyethyl)(methyl)amino]phenyl}methanone, cryptotanshinone, acetophenone, oleamide, and linoleoyl ethanolamide showed significant associations with *Prevotella*, *Anaerobutyricum*, *Ruminococcus_B*, *Dialister*, and Clostridium_P, respectively. Furthermore, L-phenylalanine, N-(5-Aminopentyl) acetamide, octopine, and acetophenone were strongly correlated with *Streptococcus* (**Figure 5C**).

## 4 Discussion

Antipsychotic-induced metabolic dysfunction is an increasingly common side effect with complex pathophysiology, which seriously impairs the health of patients with mental disorders. With the widespread use of SGAs, drug-associated metabolic disorders have garnered substantial attention. Accumulating evidence indicates that long-term SGAs treatment is associated with marked increases in body weight, as well as changes in blood glucose and blood lipid (15). A meta-analysis reported that clozapine and olanzapine confer the highest risk for body weight increase, followed by quetiapine, risperidone, paliperidone and aripiprazole (15). The results of this study are consistent with those of previous studies in term of general clinical data, including blood glucose and blood lipid related indexes (16, 17). Specifically, significant increases were observed in BMI, WC, WHR, FBG, TC, TG, IR and C-peptide following SGAs initiation. However, no statistically significant changes were detected in HDL and LDL levels before and after SGAs treatment, which may be related to the short observation period (**Table 1**). This observation aligns with the retrospective study by Hailing Cao et al., who noted that HDL alterations tend to manifest later than other metabolic markers in antipsychotic-naive patients with schizophrenia.

Emerging evidence suggests that the impact of SGAs on the gut microbiota is not a class-wide uniform effect but is influenced by the specific pharmacological profile of individual drug. For instance, the potent metabolic disruptors such as olanzapine and clozapine have been most strongly linked to significant reductions in microbial diversity and expansions in specific bacterial families like *Lachnospiraceae*, which may contribute to their high weight-gain potential (18). In contrast, drugs like aripiprazole appear to exert a milder, potentially less disruptive influence on the gut microbial signature. Furthermore, the dosage and treatment duration are likely critical factors. Although direct human studies correlating precise dosage with microbial changes are still limited, preclinical models have indicated that SGA-induced microbial shifts are dose-dependent (9), implying that higher cumulative exposure leads to more pronounced dysbiosis. Finally, medication adherence represents a significant yet often unmeasured confounding factor. Inconsistent medication intake results in a fluctuating pharmacological pressure on the gut microbiome, potentially leading to a unstable microbial profile that does not reflect a steady state, thereby complicating the interpretation of cross-sectional findings (19). We will clarify that future longitudinal studies designed to track these specific variables—drug type, dose, duration, and adherence—are essential to disentangle their individual and interactive contributions to the gut microbiome in SGA-treated patients.

Previous studies have demonstrated that antipsychotics—particularly clozapine—can alter the population diversity and composition of the gut microbiota (20). Consistently, our research found that significantly reduced α-diversity in M group compared with the B group, as evidenced by lower values of observed features and Chao1 indices. The β-diversity of the gut microbiota also had a substantial difference between two groups (**Figure 1**). It has been proven that *Bacteroidetes*, *Proteobacteria*, and *Firmicutes* were the three most dominant phyla in patients with metabolic syndrome, accounting for 92.3% of the total gut microbiota abundance (21). On the contrary, our research found that *Firmicutes_A*, *Proteobacteria*, and *Actinobacteriota* ranked in the top three abundance in B and M groups. This suggests that the gut microbiota profile of antipsychotic-naive patients initiating second-generation antipsychotics (SGAs) and exhibiting early glucose and lipid metabolism disturbances may differ from that typically observed in established metabolic syndrome. In addition, the abundance of *Proteobacteria* is correlated with obesity and related metabolic dysbiosis (22). In our cohort, *Firmicutes_A* abundance was lower in Group M than in Group B, whereas *Proteobacteria* abundance was higher—a pattern consistent with prior observations linking these shifts to metabolic perturbation (**Figure 2C**).

Lifestyle and dietary factors, smoking, and concurrent medications exert a powerful influence on the gut ecosystem. Dietary patterns, particularly the high-fat and high-sugar diets common in patients with SGA-induced weight gain, are strongly associated with reduced microbial diversity and altered short-chain fatty acid profiles (23). Smoking status is another key modifier, with evidence showing distinct microbial communities in smokers compared to non-smokers, which is highly relevant given the prevalence of smoking in psychiatric populations (24). Furthermore, polypharmacy is the rule rather than the exception. Medications commonly co-prescribed with SGAs, such as metformin for metabolic adverse effects, have been shown to reshape gut microbiota composition and function independently and significantly (25). Similarly, other psychotropic classes like antidepressants and mood stabilizers possess their own microbiota-modulating properties, creating a complex background against which SGA-specific effects must be discerned (26). Therefore, the gut microbiota in SGA-treated patients represents a dynamic interface influenced by a confluence of pharmacological, metabolic, and lifestyle factors. Our findings likely reflect the composite effect of SGAs within this multifaceted context.

At genus level, we found that the abundances of *Escherichia_710834*, *Blautia_A_141781*, *Bifidobacterium_388775* and *Faecalibacterium* were significantly different between B and M group. Specifically, the abundances of *Escherichia_710834* and *Bifidobacterium_388775* increased following first-time initiation of SGAs, whereas those of *Blautia_A_141781* and *Faecalibacterium* decreased. These findings align with previous studies showing marked increases in the abundance of *Bifidobacterium* and *Escherichia* after administration of risperidone to first-episode schizophrenia patients with normal body weight (27). It has been hypothesized that the increase in *Bifidobacterium* may represent a compensatory response to compensate for the increase in body weight and inflammatory state, which is also consistent with our findings (**Figure 2D**).

Emerging evidence indicates that early metabolic changes can occur rapidly after antipsychotic exposure, often preceding overt weight gain, and that these drugs may directly interfere with glucose metabolism pathways (28). Untargeted metabolomic profiling of gut microbial metabolites revealed pronounced shifts in the relative abundance of numerous metabolites between Group B and Group M (**Figure 4**), suggesting that SGA administration substantially reshapes the gut metabolic landscape. Notably, it is speculated that some altered metabolites are associated with glucose and lipid metabolism. For instance, oleamide—a fatty acid amide—has been shown to suppress diet-induced obesity and reduce visceral adiposity in murine models (29), while phenylalanine has repeatedly been implicated in metabolic dysregulation in human metabolomic studies (30, 31). In particular, Shuiya Sun et al. reported that patients with metabolic syndrome showed abnormal amino acid profile, and the concentrations of tyrosine, tryptophan, glutamate, phenylalanine, proline and other amino acids in plasma increased significantly (31). Intriguingly, although the serum TC, TG, HDL and LDL levels were increased after SGAs treatment (**Table 1**), gut metabolites profiling revealed a concomitant decrease in steroids and lipids, alongside a marked elevation in polypeptide products. This apparent discrepancy suggests a potential decoupling between host systemic lipid profiles and gut microbial metabolic output—a phenomenon that warrants further investigation. While numerous studies have documented amino acid dysregulation during glucose and lipid metabolism disorders (32, 33), far less is known about antipsychotic-induced alterations in microbial-derived steroids, lipids, and other small molecules. Given that the gut microbiota produces thousands of bioactive metabolites, the functional roles of most of these compounds—particularly in the context of psychopharmacological intervention—remain poorly characterized and merit systematic exploration in future studies.

The gut microbiome may influence brain function in psychiatric disorders. Barlattani et al. provided foundational evidence by showing, via MRI, that glymphatic system dysfunction is already present during acute psychotic episodes in young adults, suggesting impaired cerebral waste clearance as an early feature of psychosis (34). Building on this, Wu et al. demonstrated a direct association between gut dysbiosis and glymphatic impairment in schizophrenia patients, indicating that microbial composition is not only altered but functionally linked to the efficiency of brain waste clearance (35). The altered gut microbiome could increase intestinal permeability, leading to systemic inflammation and elevated levels of pro-inflammatory cytokines. This systemic inflammation could compromise the integrity of the blood-brain barrier and the function of astrocytic aquaporin-4 channels, which were essential for proper glymphatic flow. Consequently, the dysfunctional glymphatic system failed to effectively clear neurotoxic peptides and metabolic by-products, potentially exacerbating neuroinflammation and cognitive deficits, as evidenced by the correlation with cognitive impairment reported by Wu et al.

At the end of this study, the major metabolic pathways were found by screening landmark metabolites and enrichment analysis. These included amino acid metabolism, arginine and proline metabolism, D-amino acid metabolism, and arginine biosynthesis. Notably, ornithine emerged as a central node, showing significant involvement in all three arginine-related pathways—a finding that aligns with the observed increase in microbial peptide-derived metabolites post-treatment (**Figure 5**). Ornithine is a basic amino acid involved in urea cycle in human body and is a precursor for citrulline and proline synthesis. The relationship between ornithine and abnormal glucose and lipid metabolism has been verified by many scholars. Suzuki et al. reported a positive correlation between plasma ornithine levels and insulin resistance in humans (36), while Bhatta et al. demonstrated markedly elevated L-ornithine in a murine model of metabolic syndrome induced by a high-fat, high-sucrose (HFHS) diet over six months (37). In contrast to our findings, some studies have implicated several tryptophan-derived indole metabolites—produced by gut microbiota—in the pathogenesis of metabolic syndrome via activation of the aryl hydrocarbon receptor (AhR) pathway. We speculate that these discrepancies may stem from the distinct pharmacological effects of SGAs, which could differentially modulate host–microbiome metabolic crosstalk.

Moreover, the result showed that N-(5-Aminopentyl)acetamide was strongly correlated with *Escherichia_710834*, and oleamide and stearamide were strongly correlated with *Faecalibacterium* (**Figure 5C**). N-(5-Aminopentyl)acetamide is derived from the acetylation of the polyamine and cadaverine. Polyamine are ubiquitous in living organisms, and can bind to porin channels in the outer membrane of Gram-negative bacteria, modulating membrane permeability—suggesting a role as endogenous regulators of bacterial envelope integrity (38, 39). Oleamide and streamide are biomarkers of diabetic atherosclerotic vascular disease, implicating them in glucose metabolism dysregulation (40). *Faecalibacterium* is the core butyrate-producing genus in the human gut, contributes to intestinal homeostasis through butyrate-mediated inhibition of histone deacetylases and activation of G protein–coupled receptors (GPCRs). *Faecalibacterium* may also influence lipid amide metabolism, like oleamide and stearamide, by regulating fatty acid amide hydrolase (FAAH) (41, 42).

To our knowledge, this is the first study to apply untargeted metabolomics to characterize the gut microbial metabolites alternation following initial exposure to SGAs in patients with schizophrenia. Several limitations should be acknowledged. First, stringent inclusion criteria—restricted to medication-naive patients—limited sample size. Second, poor treatment adherence among this population resulted in a high dropout rate, necessitating a shortened observation window. Consequently, early-stage metabolic disturbances related to glucose and lipid homeostasis may not have fully manifested. Given the limited sample size, this study is exploratory in nature and underpowered to detect moderate effect sizes; findings should be validated in larger cohorts.

In summary, untargeted metabolomics analysis revealed substantial shifts in gut microbial metabolites and associated pathways before and after SGA initiation. These changes—particularly in amino acid metabolism (including arginine and proline metabolism, D-amino acid metabolism, and arginine biosynthesis)—were closely linked to drug-induced weight gain and metabolic dysfunction. Ornithine emerged as a key hub metabolite, intersecting multiple dysregulated pathways and coinciding with elevated microbial peptide derivatives post-treatment. These findings provide a mechanistic foundation for understanding antipsychotic-induced metabolic adverse effects and support future therapeutic strategies targeting the gut microbiome to mitigate glucose and lipid metabolism disorders.

## Data Availability

The 16S rRNA gene sequencing data generated in this study have been deposited in the NCBI Sequence Read Archive (SRA) under BioProject accession number PRJNA1368402: http://www.ncbi.nlm.nih.gov/bioproject/1368402. Additional metadata for each sample are available through the associated BioSample records.
